# Analysis of Segmental Lymph Node Metastasis and Clinical Features in cT1N0M0 Lung Adenocarcinoma

**DOI:** 10.1155/2020/2842604

**Published:** 2020-02-18

**Authors:** Guanghao Sun, Yanbin Sun, Zifang Zou, Shun Xu

**Affiliations:** Thoracic Surgery, China Medical University First Hospital, Shenyang, LiaoNing, China

## Abstract

The progression of lung adenocarcinoma through lymph node metastasis has been well established; however, the process of segmental lymph node (LSN) metastasis in cT1N0M0 lung adenocarcinoma remains unclear. We aimed to elucidate the markers of lymph node metastasis to different segments in early-stage lung adenocarcinoma and identify new indications for segmentectomy. A total of 200 patients were enrolled in this study. These patients were diagnosed with cT1N0M0 lung adenocarcinoma after positron emission tomography/computed tomography and received lobectomy and lymph node dissection surgeries. Lymph nodes retrieved from each station were sorted. The metastatic status of the isolated (i) LSNs and several characteristics were analyzed. Patients with ground-glass nodules (GGNs) (*P*=0.025), AIS/MIA/lepidic adenocarcinoma (*P*=0.038), nodules with a maximum diameter ≤1 cm (*P*=0.017), maximum standardized uptake value (SUV_max_) < 2.5 (*P*=0.029), serum carcinoembryonic antigen (CEA) levels ≤4.5 ng/ml (*P*=0.036), and no N1 lymph nodes metastasis (*P*=0.036) had significantly lower iLSN metastasis rates than those without these characteristics. Pure GGNs, CEA levels ≤4.5 ng/ml, SUV_max_ < 2.5, tumors with a maximum diameter of ≤1 cm, or those confirmed to be adenocarcinoma *in situ*, minimally invasive adenocarcinoma, or invasive lepidic-predominant adenocarcinoma by frozen section may indicate segmentectomy. However, segmentectomy is not suitable for patients with metastasis to the N1 lymph nodes.

## 1. Introduction

Lung cancer has the highest morbidity and mortality of any disease worldwide, including China [1, 2]. Lobectomy and mediastinal lymph node dissection have become the standard surgical treatments for non-small-cell lung cancer (NSCLC) [3], but an increasing number of researchers have found that sublobar resection, especially anatomical segmentectomy, has more advantages than and similar results to lobectomy in early-stage cases [4, 5]. Lobectomy as the standard of care has become controversial; however, two prominent studies from Japan (JCOG 0802/0804) and the United States (CALGB 140503) that compare lobectomy to sublobar resection remain inconclusive [6, 7], and these surgical methods for early-stage NSCLC remain a focus of research and discussion.

The lymphatic system is the primary pathway for lung cancer metastasis, and lymphatic metastasis is an important factor affecting the staging and prognosis of lung cancer. Before segmentectomy, one must ensure that the lymph nodes outside the tumor-bearing segments have no metastases. However, there are no clear definitions for adjacent tumor-bearing segmental nodes (aLSNs) and isolated tumor-bearing segmental nodes (iLSNs). Furthermore, due to technical difficulties and side injuries, we are unable to detect iLSNs. Therefore, when iLSNs are at high risk for metastasis, segmental resection is contraindicated [8]. However, in patients with early-stage, isolated lung adenocarcinomas, the iLSNs may be at low risk for metastasis and, therefore, anatomical segmentectomy may be a good option. Consequently, the evaluation of lymph node metastasis is of great significance for appropriate surgical planning and the recovery of postoperative patients for their long-term benefit.

This prospective study aimed to elucidate the mechanisms of metastasis to LSNs in early-stage lung adenocarcinoma. We further aimed to identify patterns of LSN metastasis and patient characteristics, including tumor size, serum tumor markers, consolidation to tumor ratio (C/T), maximum standardized uptake value (SUV_max_), and pathological subtypes, which indicate segmentectomy for cT1N0M0 lung adenocarcinoma.

## 2. Materials and Methods

### 2.1. Clinical Data

**Figure 1:**
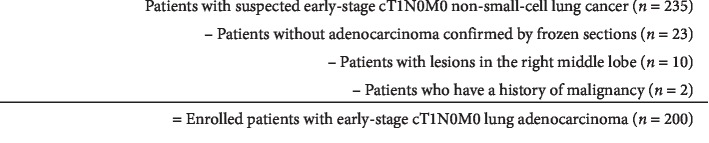
Selection procedure for all 200 enrolled cases.

A total of 235 cases of cT1N0M0 NSCLC were diagnosed by the Thoracic Surgery Department of the First Affiliated Hospital of China Medical University between January 2013 and April 2019. All patients underwent thin-slice computed tomography (CT) and a positron emission tomography/CT to confirm the clinical stage, and there was no evidence of distant metastasis. For all patients, preliminary pathological results were obtained by rapid intraoperative frozen sections before anatomical lobectomy and mediastinal lymph node dissection. Among 235 total patients, 23 were excluded because they did not have adenocarcinoma, 10 were excluded because the lesion was located in the right middle lobe, and 2 were excluded due to a history of other malignancies. The remaining 200 cases were included in the analysis.  Figure 1 shows a diagram of the patients included and excluded.

### 2.2. Lesion Removal and Sorting of Intersegmental Lymph Nodes

**Figure 2:**
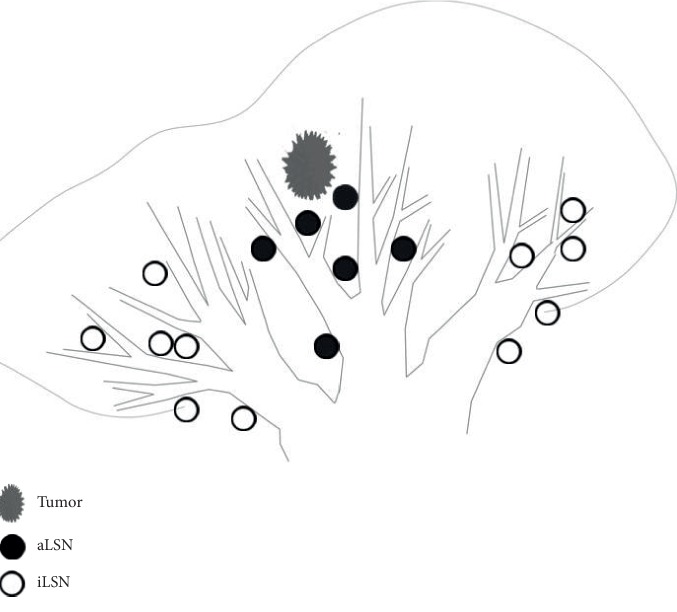
The relationship between the tumor, aLSNs, and iLSNs.

All enrolled patients underwent video-assisted thoracoscopic resection, and the ipsilateral mediastinal lymph nodes were dissected, including those in stations 2R, 3, 4R, 7, 8, 9, 10, 11, and 12 on the right and stations 4L, 5, 6, 7, 8, 9, 10, 11, and 12 on the left. After the lobes were removed, the specimens were dissected by a designated doctor. The superficial lung tissue was removed in the direction of the bronchial tree to reveal the lymph nodes between the bronchioles. According to the differences in their anatomical positions, the segmental lymph nodes (LSNs) were divided into adjacent (a) LSNs, which were proximal to the involved segmental bronchus, and isolated (i) LSNs, which were isolated from the involved segmental bronchus (Figure 2) [9]. The excised specimens and LSNs were sent to the pathology department for pathological examination. The specific pathological types and subtypes of the lesions and the metastases in each lymph node station, including aLSNs and iLSNs, were obtained.

This study was approved by the ethics committee of the First Affiliated Hospital of China Medical University. All patients provided informed consent to store their data in the Thoracic Surgery Department database for clinical research.

### 2.3. Data Analysis

In total, 200 cases of cT1N0M0 lung adenocarcinoma were included in the statistical analysis. Continuous data are expressed as means and standard deviations ($\bar{x}\pm s$), and comparisons between groups were made using Student's *t*-test. Discrete variables are expressed as composition ratios or rates. Pearson's *χ*^2^ test with continuity correction was used to perform tests of independence on the iLSN metastatic status among groups classified by imaging features (pure/part-solid ground-glass nodule (GGN) or solid nodule), serum carcinoembryonic antigen (CEA) levels (>4.5 ng/ml or ≤4.5 ng/ml), serum Cyfra 21-1 levels (>3.3 ng/ml or ≤3.3 ng/ml), SUV_max_ (≥2.5 or <2.5), pathological subtypes (adenocarcinoma *in situ* (AIS), minimally invasive adenocarcinoma (MIA), invasive lepidic-predominant adenocarcinoma (LPA), or other), maximum tumor diameter on imaging (0–1 cm, 1–2 cm, or 2–3 cm), and lymph node metastasis status (with or without N1 lymph nodes metastasis). SPSS 22.0 (IBM SPSS Statistics for Windows, IBM Corp., Version 22.0, Armonk, NY, USA) was used for statistical analysis. Logistic regression was used to identify influencing factors. *P* values <0.05 indicated statistical significance.

## 3. Results

### 3.1. Clinical and Pathological Characteristics

Table 1 shows the baseline patient and imaging characteristics, including gender, age, tumor size, preoperative serum levels of tumor markers (CEA, Cyfra 21-1, and neuron-specific enolase (NSE)), of all enrolled cases.  Table 2 shows the specific pathological subtypes of all 200 cases according to the latest adenocarcinoma classification [10].

### 3.2. Analysis of Metastasis to the Adjacent and Isolated Segmental Lymph Nodes

In total, 47 patients had N1 lymph nodes and iLSNs metastases, of which 42 had N1 lymph nodes metastasis, and 9 of 42 had not only N1 lymph nodes metastases but also iLSNs metastases; 5 patients had iLSNs metastases, all of which were confirmed to be other invasive adenocarcinomas by pathological examination and four of which had diameters of >2 cm and SUV_max_ >2.5.

We subsequently analyzed the factors influencing iLSN metastasis. Significant differences were seen in size, SUV_max_, CEA, imaging features, pathological subtype, and N1 lymph nodes metastasis by univariate analysis (Table 3). In multivariate regression analysis, we identified size, SUV_max_, CEA level, imaging features, and N1 lymph nodes metastasis as significant independent risk factors for iLSN metastasis, and AIS/MIA/LPA was identified as a significant independent protective factor against iLSN metastasis (Table 4). Therefore, patients without N1 lymph node metastasis (*P*=0.036), with AIS/MIA/LPA (*P*=0.038), SUV_max_ < 2.5 (*P*=0.029), serum CEA levels ≤4.5 ng/ml (*P*=0.036), purely GGN lesions (*P*=0.025), or a maximum tumor diameter of ≤1 cm (*P*=0.017) had significantly lower iLSN metastasis rates than those without these characteristics.

## 4. Discussion

In 1995, a randomized controlled trial by the Lung Cancer Research Group that compared lobectomy and limited resection for early-stage NSCLC found that patients who received limited resection had a 75% increase in recurrence rate (*P*=0.02, one-sided), a 50% increase in cancer mortality (*P*=0.09, one-sided), and a 30% increase in overall mortality (*P*=0.08, one-sided) compared to patients who underwent lobectomy. Since then, lobectomy and mediastinal lymph node dissection have become the standard surgical treatment for early-stage NSCLC [3]. However, the conclusions of that study have many limitations. For example, in the sublobar resection group, approximately one-third of the patients were treated with partial resection, which is not based on anatomical structure. Furthermore, there were no routine CT scans, which could have affected the tumor measurements [11]. These limitations would have had a significant impact on the results of the analysis. Recent studies have shown that, for patients with early-stage lung adenocarcinoma, sublobar resection, including anatomical segmentectomy, has the advantages of less postoperative chest drainage, shorter catheterization time, and greater preservation of healthy lung tissue compared to lobectomy. Furthermore, retrospective studies have shown that, in patients with isolated lung adenocarcinomas ≤2 cm in diameter, anatomical segmentectomy for NSCLCs other than large cell carcinoma can achieve local recurrence and postoperative long-term survival rates comparable to those of standard radical lung cancer surgeries [12].

For lung adenocarcinoma, lymphatic drainage usually flows from the inside to the outside, from the near to the distant, and from the intrapulmonary lobe to the hilar to the mediastinal lymph nodes [13]. Therefore, because the 12th, 13th, and 14th stations are located near the lesion, the 13th and 14th station lymph nodes, especially, may be in the same segment as the lesion and may be metastasized first. In our study, the incidence of 13th and 14th station lymph node metastasis was 23.5%; however, after sorting the LSNs, only 7% of patients had affected iLSNs. No definitive patterns of LSN metastasis have been published to date; yet, one must ensure that adjacent lymph nodes in the same segment as the lesion are metastasis-free before attempting a segmentectomy. Therefore, we aimed to identify independent predictive factors for iLSN metastasis to identify patients unsuitable for segmentectomy and enable patients to receive the appropriate treatment.

Studies have shown that serum tumor markers may be an independent risk factor for early-stage lung adenocarcinoma [14]. In our study, we analyzed the associations between multiple tumor markers and lymph node metastasis. However, in the univariate analysis, CEA had a significant association with iLSN metastasis; Cyfra 21-1 and NSE had no significant association. In the subsequent multivariate analysis, serum CEA levels were identified as independent risk factors for early-stage lymph node metastasis in lung adenocarcinoma. These results indicate that patients whose serum CEA levels were <4.3 ng/ml may be suitable to receive segmentectomy. Cyfra 21-1 and NSE may not have been identified as risk factors because increases in Cyfra 21-1 and NSE are primarily caused by squamous cell carcinoma and small-cell lung cancer, respectively [15]. However, in this study, we observed a trend of increased Cyfra 21-1 and NSE in patients with metastatic LSNs compared to those without metastatic LSNs. This association may require a larger sample to confirm.

Although it is controversial, the latest edition of the National Cancer Center Network guidelines states that tumors with a maximum diameter of <2 cm are an indication for segmental resection. Similarly, Matsurama et al. showed that, in patients with NSCLC with a maximum peripheral diameter of <2 cm, the resection range included the corresponding segmental, hilar, and mediastinal lymph nodes, and iLSNs were not necessary for detection [16]. In our study, we used the maximum tumor diameter as a continuous variable rather than a discrete variable for analysis. We found that the maximum tumor diameter and the probability of lymph node metastasis between the segments were positively correlated. We further performed a regression analysis with the maximum tumor diameter as a grouping variable (Table 5). We found that the probability of iLSN metastasis in tumors with a maximum diameter of 2-3 is 30 times that of tumors with a maximum diameter of 0-1, and the probability of iLSN metastasis in tumors with a maximum diameter of 1-2 is twice that of tumors with a maximum diameter of 0-1. This further demonstrates that tumors with a maximum diameter of <2 cm may be an indication for segmentectomy, and the smaller the tumor, the safer it may be.

SUV_max_ is a semiquantitative indicator commonly used in the diagnosis of tumors. It refers to the ratio of the activity of the imaging agent in the local tissue to that in the entire body and can reflect the activity in tumor cells to some extent. Studies have shown that the primary 18F-fluorodeoxyglucose metabolism of lung adenocarcinoma is related to hilar and mediastinal lymph node metastasis. The lymph node metastasis rate increases with an increase in tumor SUV_max_ [17]; thus, we expect it may also be associated with the metastasis of LSNs. It is well known that normal lung tissue has a SUV_max_ of 2, and when the SUV_max_ exceeds 2.5, we consider the lung tissue to be malignant. However, in actual clinical work, some tumors have a SUV_max_ between 2 and 2.5, or even <2. Postoperative pathology is also an indicator of early-stage adenocarcinoma; thus, we used 2.5 as a cutoff value in our analysis, similar to that used in a related study [18]. In our study, the probability of iLSN metastasis in patients with a baseline SUV_max_ of >2.5 was 9 times that of patients whose SUV_max_ did not exceed 2.5. Thus, we conclude that the lower the SUV_max_, the lower the chance of metastasis.

Imaging data is an essential component of preoperative examination data, in addition to the results of blood tests. Lung adenocarcinoma accounts for a large proportion of solitary pulmonary nodules. Depending on the proportion of the solid component, we can divide these nodules into pure GGNs, partially solid nodules, and solid nodules. According to the latest classification criteria for lung adenocarcinoma, lung adenocarcinoma can be divided into different subtypes. According to the strength of invasiveness, lung adenocarcinoma can be divided into two groups: strong (A) and weak (B). Group A includes AIS, MIA, and LPA, despite their genetic differences; however, regarding biological behavior, they can be classified as the less aggressive of the two groups [19]. These adenocarcinomas usually appear on CT as pure GGNs. If the lesion is completely removed, the disease-free survival rate approaches 100% [20]. This further demonstrates that segmentectomy with maximal lung tissue retention is more suitable for patients with group A adenocarcinomas. Group B is more invasive than group A and, thus, is not suitable for segmentectomy [21]. This is consistent with the results of our research. In pure GGNs or nodules with AIS/MIA/LPA, iLSNs had a lower probability of metastasis than those without GGNs or LPA and may be more suitable for segmental resection.

We found that 9 of the 33 cases with N1 lymph node metastases had iLSN metastases. After further statistical analysis, we found that N1 metastasis was an independent risk factor for iLSN metastasis. This provides a new way to evaluate whether lobectomy is indicated during surgery. If intraoperative N1 lymph node frozen sections are positive, lobectomy should be performed.

### 4.1. Limitations

This study had some limitations. First, the sample size was small because the probability of aLSN metastasis is relatively low in early-stage and, therefore, the number of cases with iLSN metastasis was minimal. Furthermore, all patients underwent lobectomy, which had a selective shift. Therefore, the results of this study still require a larger sample, and further studies by more centers are necessary for further confirmation.

In addition, the SUV_max_ value may only represent the activity levels of the lesion at a particular time or a certain period of time before surgery and does not correctly represent the activity and invasiveness of the overall lesion, although we have obtained favorable results in the study. However, the extent of the impact of SUV_max_ on iLSN metastasis is open to interpretation and may even require multiple measurements to obtain a more precise conclusion.

Finally, although studies have shown that intraoperative frozen section pathological diagnosis is a fast and effective method [22], the accurate evaluation of intraoperative pathology is controversial [23]. Furthermore, this always presents a challenge for the surgeon, the person who performs the section, and the pathologist.

## 5. Conclusions

Our aim was to provide criteria for the indication of segmentectomy and identify markers for patients with no iLSN metastasis for the promotion of precision and targeted medicine. We found that patients with pure GGNs, CEA ≤ 4.5 ng/ml, SUV_max_ < 2.5, or a maximum tumor diameter of ≤1 cm, whose intraoperative frozen section was confirmed as AIS/MIA/LPA, or whose N1 lymph nodes frozen sections were negative had a low probability of iLSN metastasis and, thus, may be suitable for segmentectomy.

## Figures and Tables

**Table 1:** Clinical data from 200 cases.

Clinical date	Number
Gender	
Male	102
Female	98
Age (years)	58.435 ± 5.735
Serum CEA level	
≤4.5 ng/ml	153
>4.5 ng/ml	47
Serum Cyfra 21-1 level	
≤3.3 ng/ml	168
>3.3 ng/ml	32
Serum NSE level	
≤16.30 ng/ml	87
>16.30 ng/ml	113
SUV_max_	
<2.5	117
≥2.5	83
Tumor size	
0–1 cm	95
1–2 cm	78
2–3 cm	27
Imaging features	
Pure GGN	125
Part-solid nodule	40
Solid nodule	35

**Table 2:** Postoperative pathological results in 200 patients.

Pathological subtype	Number
Adenocarcinoma *in situ*	29
Minimally invasive adenocarcinoma	40
Invasive lepidic-predominant adenocarcinoma	50
Others	81

**Table 3:** Univariate analysis of iLSN metastases in 200 cases.

Influence factor	Without iLSN metastasis (*n* = 186)	With iLSN metastasis (*n* = 14)	t/*χ*^2^	*P* value
Age (years)	58.94 ± 7.37	57.93 ± 3.38	0.955	0.349
Gender			1.063	0.302
Male	93 (50.0)	9 (64.3)		
Female	93 (50.0)	5 (35.7)		
Serum Cyfra 21-1 level			0.000	1.000
≤3.3 ng/ml	156 (83.9)	12 (85.7)		
>3.3 ng/ml	30 (16.1)	2 (14.3)		
Serum NSE level			2.984	0.084
≤16.30 ng/ml	84 (45.2)	3 (21.4)		
>16.30 ng/ml	102 (54.8)	11 (78.6)		
Serum CEA level			5.88	0.015
≤4.5 ng/ml	146 (78.5)	7 (50.0)		
>4.5 ng/ml	40 (21.5)	7 (50.0)		
Tumor size			54.736	<0.001
0–1	94 (50.5)	1 (7.1)		
1–2	76 (40.9)	2 (14.3)		
2–3	16 (8.6)	11 (78.6)		
SUV_max_			8.522	0.004
<2.5	114 (61.3)	3 (21.4)		
≥2.5	72 (38.7)	11 (78.6)		
Imaging features			19.683	<0.001
Pure GGN	124 (66.7)	1 (7.1)		
Nonpure GGN	62 (33.3)	13 (92.9)		
Pathological subtype			12.771	<0.001
Other IA	69 (37.1)	12 (85.7)		
AIS/MIA/LPA	117 (62.9)	2 (14.3)		
N1 lymph nodes metastasis			14.312	<0.001
No	153 (82.3)	5 (35.7)		
Yes	33 (17.7)	9 (64.3)		

**Table 4:** Multivariate logistic analysis of iLSN metastases in 200 cases (1).

Factor	*B*	SE	Wald	*P* value	OR	95% CL	
						Lower limit	Upper limit
Tumor size	1.978	0.830	5.683	0.017	7.231	1.422	36.782
SUV_max_	2.210	1.012	4.774	0.029	9.116	1.256	66.194
CEA	2.593	1.236	4.400	0.036	13.368	1.185	150.768
Imaging features	2.796	1.251	4.996	0.025	16.377	1.411	190.106
AIS/MIA/LPA	−2.728	1.312	4.323	0.038	0.065	0.005	0.855
N1 lymph nodes metastasis	2.093	1.000	4.380	0.036	8.109	1.142	57.577

**Table 5:** Multivariate logistic analysis of iLSN metastases in 200 cases (2).

Factor	*B*	SE	Wald	*P* value	OR	95% CL	
						Lower limit	Upper limit
Size							
0–1			6.828	0.033			
1–2	0.327	1.556	0.044	0.834	1.387	0.066	29.261
2–3	3.409	1.572	4.701	0.030	30.224	1.387	658.446
SUV_max_	2.210	1.012	4.774	0.029	9.116	1.256	66.194
CEA level	2.593	1.236	4.400	0.036	13.368	1.185	150.768
Imaging features	2.796	1.251	4.996	0.025	16.377	1.411	190.106
AIS/MIA/LPA	−2.728	1.312	4.323	0.038	0.065	0.005	0.855
N1 lymph nodes metastasis	2.093	1.000	4.380	0.036	8.109	1.142	57.577

## Data Availability

The data used to support the findings of this study are available from the corresponding author upon request.

## References

[1] Freddie B., Jacques F., Isabelle S. (2018). Global cancer Statistics 2018: GLOBOCAN estimates of incidence and mortality worldwide for 36 cancers in 185 countries. *A Cancer Journal for Clinicians*.

[2] Zhang S. W., Zheng R. S., Yang Z. X. (2018). Trend analysis on incidence and age at diagnosis for lung cancer in cancer registration areas of China, 2000-2014. *Zhonghua Yu Fang Yi Xue Za Zhi*.

[3] Ginsberg R. J., Rubinstein L. V. (1995). Randomized trial of lobectomy versus limited resection for T1 N0 non-small cell lung cancer. *The Annals of Thoracic Surgery*.

[4] Zhang Z., Zhang Y., Feng J. H., Yao D. Z., Liu J., Wei D. (2013). Is video-assisted thoracic surgery lobectomy better than thoracotomy for early-stage non-small-cell lung cancer? A systematic review and meta-analysis. *European Journal of Cardio-Thoracic Surgery*.

[5] Okumura M., Goto M., Ideguchi K. (2007). Factors associated with outcome of segmentectomy for non-small cell lung cancer: long-term follow-up study at a single institution in Japan. *Lung Cancer*.

[6] Nakamura K., Saji H., Nakajima R. (2010). A phase III randomized trial of lobectomy versus limited resection for small-sized peripheral non-small cell lung cancer (JCOG0802/WJOG4607L). *Japanese Journal of Clinical Oncology*.

[7] Fox N., Bauer T. (2008). Calgb 140503: a randomized phase III trial of lobectomy versus sublobar resection for small. *Oncology Issues*.

[8] Sakairi Y., Yoshino I., Yoshida T. S., Suzuki T. H., Mizobuchi T., Iwata T. (2014). Pattern of metastasis outside tumor-bearing segments in primary lung cancer: rationale for segmentectomy. *The Annals of Thoracic Surgery*.

[9] Wang X., Yan S., Lv C. (2017). Impact of omission of intrapulmonary lymph node retrieval on outcome evaluation of lung cancer patients without lymph node metastasis: a propensity score matching analysis. *Clinical Lung Cancer*.

[10] Travis W. D., Brambilla E., Noguchi M. (2011). International association for the study of lung cancer/American thoracic society/European respiratory society: international multidisciplinary classification of lung adenocarcinoma: executive summary. *Proceedings of the American Thoracic Society*.

[11] Sassine G., Sandy E. B., Sami H., Weerasinghe C., Atallah J. P. (2017). What we know about surgical therapy in early-stage non-small-cell lung cancer: a guide for the medical oncologist. *Cancer Management and Research*.

[12] El-Sherif A., Gooding W. E., Santos R. (2006). Outcomes of sublobar resection versus lobectomy for stage I non–small cell lung cancer: a 13-year analysis. *Annals of Thoracic Surgery*.

[13] Naruke T. (1978). Lymph node mapping and curability at various levels of metastasis in resected lung cancer. *The Journal of Thoracic and Cardiovascular Surgery*.

[14] Grunnet M., Sorensen J. B. (2012). Carcinoembryonic antigen (CEA) as tumor marker in lung cancer. *Lung Cancer*.

[15] Liu X., Zhang W., Yin W. (2017). The prognostic value of the serum neuron specific enolase and lactate dehydrogenase in small cell lung cancer patients receiving first-line platinum-based chemotherapy. *Medicine*.

[16] Matsumura Y., Hishida T., Yoshida G. J., Aokage K., Ishii G., Nagai K. (2012). Reasonable extent of lymph node dissection in intentional segmentectomy for small-sized peripheral non-small-cell lung cancer: from the clinicopathological findings of patients who underwent lobectomy with systematic lymph node dissection. *Journal of Thoracic Oncology*.

[17] Li M., Wu N., Zheng R. (2013). Primary tumor PET/CT [F-18]FDG uptake is an independent predictive factor for regional lymph node metastasis in patients with non-small cell lung cancer. *Cancer Imaging*.

[18] Lyu L., Liu Y., Wang X. Y. (2019). Potential value of FDG PET-CT in predicting occult lymph node metastasis in clinical stage IA lung adenocarcinoma. *Zhonghua Zhong Liu Za Zhi*.

[19] Kadota K., Villena-Vargas J., Yoshizawa A. (2014). Prognostic significance of adenocarcinoma in situ, minimally invasive adenocarcinoma, and nonmucinous lepidic predominant invasive adenocarcinoma of the lung in patients with stage I disease. *The American Journal of Surgical Pathology*.

[20] Lee H. Y., Choi Y.-L., Lee J. I. K. S., Han J. W. J., Moon J. I., Shim Y. M. (2014). Pure ground-glass opacity neoplastic lung nodules: histopathology, imaging, and management. *American Journal of Roentgenology*.

[21] Zombori T., Nyári T., Tiszlavicz L. (2018). The more the micropapillary pattern in stage I lung adenocarcinoma, the worse the prognosis—a retrospective study on digitalized slides. *Virchows Archiv*.

[22] Xu X., Chung J.-H., Jheon S., Sung C.-T. S. W., Choe G., Lee J. H. (2010). The accuracy of frozen section diagnosis of pulmonary nodules: evaluation of inflation method during intraoperative pathology consultation with cryosection. *Journal of Thoracic Oncology*.

[23] Bittar H. E. T., Incharoen P., Althouse A. D., Dacic S. (2015). Accuracy of the IASLC/ATS/ERS histological subtyping of stage I lung adenocarcinoma on intraoperative frozen sections. *Modern Pathology*.

